# Ultrasound-Assisted Aqueous Extraction of Chlorogenic Acid and Cynarin with the Impact of Inulin from Burdock (*Arctium lappa* L.) Roots

**DOI:** 10.3390/antiox11071219

**Published:** 2022-06-22

**Authors:** Yuan Chen, Jing-Yi Su, Chun-Yao Yang

**Affiliations:** Department of Food Science, Fu Jen Catholic University, No. 510, Zhongzheng Rd., Xinzhuang District, New Taipei City 242062, Taiwan; yuan110124@gmail.com (Y.C.); f12294561155@gmail.com (J.-Y.S.)

**Keywords:** burdock roots, ultrasound-assisted extraction, chlorogenic acid, cynarin, inulin

## Abstract

The ultrasound-assisted aqueous extraction of chlorogenic acid (CGA) and cynarin with the impact of inulin from burdock (*Arctium lappa* L.) roots was investigated. Three extraction modes, ultrasound at 40 kHz/300 W (U-40), ultrasound at 120 kHz/300 W (U-120), and shaking at 120 rpm (S-120), were compared. The effects of process parameters on the extraction of polyphenols, CGA, cynarin, inulin, and antioxidant activity using U-40 were evaluated. In 10 min, 50 °C, and 1/30 (g/mL-water) of solid-to-liquid ratio, the order of CGA content in the dried burdock root powder (DBR) was U-40 (484.65 μg/g-DBR) > U-120 (369.93 μg/g-DBR) > S-120 (176.99 μg/g-DBR), while the order of cynarin content in DBR was U-120 (376.47 μg/g-DBR) > U-40 (341.54 μg/g-DBR) > S-120 (330.44 μg/g-DBR), showing the selective extraction of CGA and cynarin between using 40 and 120 kHz of ultrasound. The profiles of increase and then decrease in free CGA and cynarin concentrations against time revealed their degradation, including their interactions with the abundant inulin. The kinetic model, considering extraction followed by degradation, was proposed to describe the variations of free CGA and cynarin against time. This study provides an effective method using water to extract CGA, cynarin, and inulin from burdock roots.

## 1. Introduction

Burdock (*Arctium lappa* L.) is a medicinal plant used in traditional medicine for the treatment in vivo (diuresis, hypoglycemia) and in vitro (eczema, skin infection) [1], and burdock roots have been consumed as food in many countries in Asia and Europe [2,3]. Burdock roots contain flavonoids, polyphenols, polysaccharides, and polyunsaturated fatty acids with biological and pharmacological functions, such as antioxidant, anti-inflammatory, anti-diabetic, anti-carcinogenic, anti-allergic, anti-ulcer, gastroprotective, hepatoprotective, anti-aging, and anti-cytotoxic effects [4]. The significant constituents in the burdock roots are polysaccharides with high-levels of inulin as the dietary fiber [3,5] and polyphenolic compounds mainly being caffeoylquinic acids, such as chlorogenic acid (CGA, 3-O-caffeoylquinic acid) and cynarin (1,5-dicaffeoylquinic acid) [6,7,8]. CGA possesses a high antioxidant activity with health benefits for human, including antihypertensive, antidiabetic, antineurodegenerative, and antilipidemic activities [9,10,11], cynarin shows the function of intervening against glycoxidation [12], and inulin behaves positive effects on blood glucose attenuation, lipid homeostasis, mineral bioavailability and immunomodulation [13]. Hence, those bioactive compounds are worthwhile to be extracted from burdock roots in a significant content, and can be served as the nutritional supplement in foods. However, the compositions of the main bioactive compounds in burdock roots and the nutritional effect are generally subjected to the various varieties and the cultivated conditions [7].

For the production of burdock root products, the profiles of bioactive compounds are affected by the processing method and conditions, such as the volatile compounds of burdock root tea that are affected by the drying methods [14]. In addition, the interaction of phenolic acids with the polysaccharides in the burdock roots could occur during the processing. Passos et al. (2021) corroborated the existence of CGA bound to polysaccharides under higher carbohydrate content, with CGA not detected in free form [15]. In the burdock roots, the content of polysaccharide inulin varies much in the range of 12–17% of the fresh burdock roots (or 50–70% of dried weight) [16]. Thus, when performing the processing or extraction of bioactive compounds from burdock roots, the caffeoylquinic acids could have the possibility to degrade from the interaction with the abundant inulin by phenolic binding to form the complex during the progress of extraction. In addition, under the influence of temperature, pH, light, and heating time, the caffeoylquinic acids in the aqueous solutions will degrade or isomerize via the degradation pathways of isomerization, methylation, and hydrolysis [17,18,19,20]. Hence, it is beneficial to develop an efficient extraction process that can extract these bioactive compounds in high contents from burdock roots and reduce the extent of thermal degradation and possible interactions of the phenolic acids with the abundant inulin. Ultrasound-assisted extraction under suitable frequency and conditions could be the favorable choice for the extraction purpose.

Ultrasound with frequency waves above 20 kHz can promote chemical reactions by generating cavities and local hotspots in the liquid medium, and has been employed in food processing efficiently [21,22,23]. Ultrasound can be used to extract the bioactive compounds from plant materials by destroying the plant cell walls to facilitate the transport of ingredients and increase the extraction efficiency [24,25,26]. For the solvent extraction of bioactive compounds from plant materials, the polarity of the solvent plays an important role in the extraction efficiency. Moreover, the interaction between the extracted components can affect the profile of the constituents in the extract. In this study, water was chosen as the green solvent to extract the bioactive compounds from burdock roots of the Yanagawarisou variety, due to its cheapness, nontoxicity, easy separation from the product, high solubility for polysaccharides and polyphenols with more hydrophilicity. Using ultrasound to promote the extraction of phenolic acids, the ultrasonic frequency and extraction time probably have significant effects on the selective extraction of CGA and cynarin in the presence of abundant inulin from burdock roots. Therefore, the aim of the study was to investigate the ultrasound-assisted aqueous extraction of polyphenol, free CGA, and cynarin from burdock roots of the Yanagawarisou variety with the impact of inulin simultaneously extracted. The effects of ultrasonic frequencies of 40 kHz and 120 kHz and the shaking extraction at 120 rpm on the extraction efficiency of bioactive compounds were evaluated. The effects of the process parameters, solid-to-liquid ratio, temperature, and extraction time on the variations of free CGA, cynarin, inulin, and total phenolic content, as well as antioxidant activity, were explored. The kinetic model of first-order series reactions was proposed for free CGA and cynarin in the aqueous solution to describe their extraction followed by their degradation, including thermal degradation and interactions with the polysaccharide inulin, and the related kinetic parameters were correlated.

## 2. Materials and Methods

### 2.1. Materials

The burdock (*Arctium lappa* L.) roots of the Yanagawarisou variety that were harvested in April 2020, were purchased from Jiali District Farmers’ Association in Tainan City, Taiwan (R.O.C.). The chemical reagents, (±)-6-hydroxy-2,5,7,8-tetramethylchromane-2-carboxylic acid, 2,4,6-tris(2-pyridyl)-s-triazine, 3,5-dinitrosalicylic acid, Folin & Ciocalteu’s phenol reagent, gallic acid, ferrous sulfate heptahydrate, and ferric chloride were purchased from Sigma-Aldrich Co. (St. Louis, MO, USA). The purchased reagents used as standard in analysis were chlorogenic acid from Sigma-Aldrich Co. (St. Louis, MO, USA), cynarin and inulin from Toronto Research Chemicals Inc. (Toronto, ON, Canada), and fructose from Chem Service Inc. (West Chester, PA, USA). Other chemicals were purchased from Bionovas biotechnology Co. (Toronto, ON, Canada), Merck (Darmstadt, Germany), Sigma-Aldrich Co. (St. Louis, MO, USA), and IT’S Science Corporation Ltd. (New Taipei City, Taiwan).

### 2.2. Preparation of the Dried Burdock Root Powder

The burdock root purchased was first treated by removing the fibrous root from the surface, then was washed thoroughly and sliced. The water content in the fresh burdock root (denoted as BR) was determined as being 76.88 ± 0.43 g-water/100 g-BR (*n* = 6) [27], which was close to the published data [5]. Prior to the extraction operation, the fresh burdock root was further freeze-dried to remove water. After that, the dried burdock root was ground and screened with 60-mesh sieves to obtain the dried burdock root powder (denoted as DBR), which was preserved away from light at 4 °C and used as the raw material in the extraction process.

### 2.3. Ultrasound-Assisted Extraction and Shaking Extraction

The extraction of bioactive compounds from DBR was performed by using ultrasonic bath systems with 40 kHz/300 W or 120 kHz/300 W of ultrasound frequency/electric power (LEO-3002S, LEO-3002H, LEO Ultrasonic Co., New Taipei City, Taiwan), and the powder density of the ultrasonic system was 0.028 W/mL. The definite quantity of DBR was introduced into a 250 mL flask that contained the de-ionized water solvent in the selected ratio of solid (g-DBR) to liquid (mL-water) (1/10, 1/20, 1/30, or 1/40 g-DBR/mL-water) at the setting temperature (30, 40, 50, or 60 °C). The extraction process was started with ultrasound irradiation of 40 kHz/300 W (denoted as U-40) or 120 kHz/300 W (denoted as U-120), and the extraction without ultrasound but with reciprocal shaking bath (Model B602D, Firstek, Taipei, Taiwan) at 120 rom (denoted as S-120) was carried out for comparison. At the selected extraction time (0–90 min), the aqueous phase containing the polyphenolic compounds and polysaccharides was separated from the solid–liquid mixture by centrifugation at 3100 g (4000 rpm) for 10 min. Then, the water solvent was removed from the aqueous solution by freeze-drying to obtain the dried burdock root extract (denoted as BRE). The yield of the extract from DBR was estimated by using Equation (1):

Extract yield (% of DBR) = (weight of BRE/weight of DBR) × 100%(1)

### 2.4. Determination of Total Phenolic Content

The determination of total phenolic content (TPC) in BRE followed the method of Kujala et al. (2000), with a modification using a gallic acid equivalent (denoted as GAE) [28]. The prepared liquid sample of BRE (375 μL) was well mixed with 375 μL of 1 N Folin-Ciocalteu reagent at room temperature, and left to react for 5 min. Then, 750 μL of Na_2_CO_3_ (20%) was added into the above mixture to react for 8 min, and the absorbance of the reaction mixture was measured using a spectrophotometer at 730 nm (Hitachi, Ratio Beam Spectrophotometer U-5100, Tokyo, Japan) to determine the TPC of the BRE sample, which was expressed as mg-GAE/g-DBR by multiplying mg-GAE/g-BRE with g-BRE/g-DBR.

### 2.5. Determination of Ferric-Reducing Antioxidant Power

Ferric-reducing antioxidant power (FRAP) was applied to evaluate the antioxidant activity of BRE by following the method of Chen and Yang (2020), with a slight modification using a ferrous sulfate heptahydrate equivalent (denoted as FSE) [29]. The FRAP reagent was prepared and kept at 37 °C for use. Then, the prepared liquid sample of BRE (15 μL), FRAP reagent (285 μL), and water (500 μL) was mixed to react at 37 °C for 4 min. The FRAP of the BRE sample was determined by measuring the absorbance of the mixture at 593 nm, and was expressed as mg-FSE/g-DBR.

### 2.6. Determination of Inulin Content

The quantification of inulin content in the plant extract includes direct methods and indirect methods, where indirect spectrophotometric methods have been developed for the determination of the inulin content as the difference between total carbohydrate and reducing sugars [13,30,31]. In this study, the inulin content in BRE was determined with the indirect method by following the method of Milani et al. (2011) using the equation: Inulin content (%) = Total carbohydrate content (%) − Total reducing sugar (%) [13]. The total reducing sugar in BRE was determined by following the 3,5-dinitrosalicylic acid (DNS) method with a slight modification, using fructose as the standard [32]. The measurement of total carbohydrate content in BRE followed the phenol–sulfuric acid method with a slight modification using inulin as the standard, and was performed at 490 nm of wavelength using a spectrophotometer [33].

### 2.7. HPLC Analysis of Chlorogenic Acid and Cynarin

The contents of the free form of chlorogenic acid (CGA) and cynarin in BRE that contained inulin were determined by using high performance liquid chromatography (HPLC). The long chain inulin can be precipitated from aqueous solutions using high concentrations of organic solvents such as methanol, ethanol, and propanol [30]. Thus, to prepare the liquid sample of BRE for the HPLC analysis of free CGA and cynarin, a definite amount of BRE was added into methanol, and the suspended precipitates were formed in the liquid. These suspended precipitates in the liquid were removed by centrifugation, and the supernatant liquid sample was filtered with 0.45 μm membrane filters prior to its injection into the HPLC system. The method of Ferracane et al. (2010) was followed with a slight modification, using standards of CGA and cynarin for HPLC analysis [34]. The HPLC system was equipped with a Mightysil RP-18 GP column (5 μm, 250 mm × 4.6 mm, Kanto Chemical Co., Tokyo, Japan) operated at room temperature with a diode array detector (HITACHI Chromaster 5430, Tokyo, Japan), set at 280 nm of wavelength, and the injection volume was 20 μL. The mobile phase was solvent A (acetonitrile/methanol at 60:40 (*v*/*v*)) and solvent B (0.1% trifluoroacetic acid) at the flow rate of 0.8 mL/min with the gradients set as solvent A: 20 to 30% at 0 to 6 min, 30 to 40% at 6 to 16 min, 40 to 50% at 16 to 24 min, 50 to 90% at 24 to 32 min, 90% at 32 to 35 min, and 90 to 20% at 35 to 41 min.

### 2.8. Analysis of Morphological Structures

The surface morphologies of dried burdock root powder before extraction and its extraction residues, obtained using an ultrasound-assisted extraction at different frequencies and shaking extraction without ultrasound, were verified by a field emission scanning electron microscope (FESEM) (JEOL JSM-7800F, Tokyo, Japan).

### 2.9. Statistical Analysis

The experimental data are expressed as mean ± standard deviations from triplicate experiments with three independent samples (*n* = 3). Statistical analysis was evaluated by one-way ANOVA with the Duncan’s multiple range test using IBM SPSS Statistics 20 (IBM SPSS Statistics for Windows v. 20.0, IBM Corp, Armonk, NY, USA), and the statistically significant difference was determined at *p* < 0.05.

### 2.10. Modelling for Extraction Kinetics

The caffeoylquinic acids in the aqueous solutions will degrade or isomerize via degradation pathways under the effects of temperature, pH, light, and heating time [17,18,19,20]. In the present study, the burdock roots contain abundant polysaccharide inulin that may interact with the free CGA and cynarin by phenolic binding to conduct complexation. In addition, the free CGA and cynarin in the aqueous phase might undergo different extents of thermal degradation. Accordingly, during the extraction, the extracted free CGA or cynarin is proposed to be affected by thermal degradation and complexation by phenolic binding to polysaccharides, and these two effects can be combined as a degradation reaction occurring in the aqueous phase.

Therefore, the transport of free form of CGA (or cynarin) extracted from the solid DBR into the water phase involves several steps, i.e., (1) the dissolution and diffusion of CGA (or cynarin) into the aqueous phase as the free compound with the apparent rate coefficient *k*, and (2) degradation of the free CGA (or cynarin) into the degraded product with the apparent rate coefficient *k_d_*. The extraction and degradation of CGA (or cynarin) are described by the first-order series reactions in the following scheme:

$$C_{S}\;\left(\mathrm{inside}\;\mathrm{solid}\right)\overset{k}{\to }C_{A}\;\;\left(\mathrm{in}\;a\;\mathrm{queous}\;\mathrm{phase}\right)\overset{k_{d}}{\to }P\;\left(\mathrm{degraded}\;\mathrm{product}\right)\;$$

The amount of extractable CGA (or cynarin) inside the dried burdock root under specific conditions during the extraction time is designated as *C_S_* (*t*), the amount of free CGA (or cynarin) that has been extracted into the aqueous phase is represented as *C_A_* (*t*), and the degraded product is expressed as *P* (*t*). The rates of expression of *C_S_* (*t*), *C_A_* (*t*), and *P* (*t*) are

*dC_S_* (*t*)/*dt* = −*kC_S_* (*t*), (2)

*dC_A_* (*t*)/*dt* = *kC_S_* (*t*) − *k_d_C_A_* (*t*), (3)

*dP (t)/dt = k_d_C_A_ (t),* (4)

with the initial conditions *C_S_* (0) = *C_E_*, *C_A_* (0) = 0, and *P* (0) = 0, where *C_E_* represents the total extractable CGA (or cynarin) within the dried burdock root under specific conditions and *C_E_* = *C_S_* (*t*) + *C_A_* (*t*) + *P* (*t*). Then, Equation (2) is solved to obtain

*C_S_* (*t*) = *C_E_* exp(−*kt*). (5)

Equation (5) is substituted into Equation (3) and rearranged to get

*dC_A_* (*t*)/*dt* + *k_d_C_A_* (*t*) = *kC_E_* exp(−*kt*). (6)

Equation (6) is solved to give

*C_A_* (*t*) = *C_E_* [*k*/(*k_d_* − *k*)] [exp(−*kt*) − exp(−*k_d_t*)]. (7)

Equation (7) describes the existence of a maximum value of *C_A_* (*t*). The maximum concentration *C_A_*_,max_ at the time *t*_max_ can be derived as

*t_max_* = ln(*k*/*k_d_*)/(*k* − *k_d_*), (8)

ln(*C_A_,_max_*) = ln(*C_E_*) + [*k_d_*/(*k_d_* − *k*)] ln(*k*/*k_d_*). (9)

From the experimental data, the parameters *k*, *k_d_*, and *C_E_* can be correlated by applying the nonlinear least square method using Equations (7) and (9).

## 3. Results and Discussion

### 3.1. Effect of Extraction Modes

**Figure 1:**
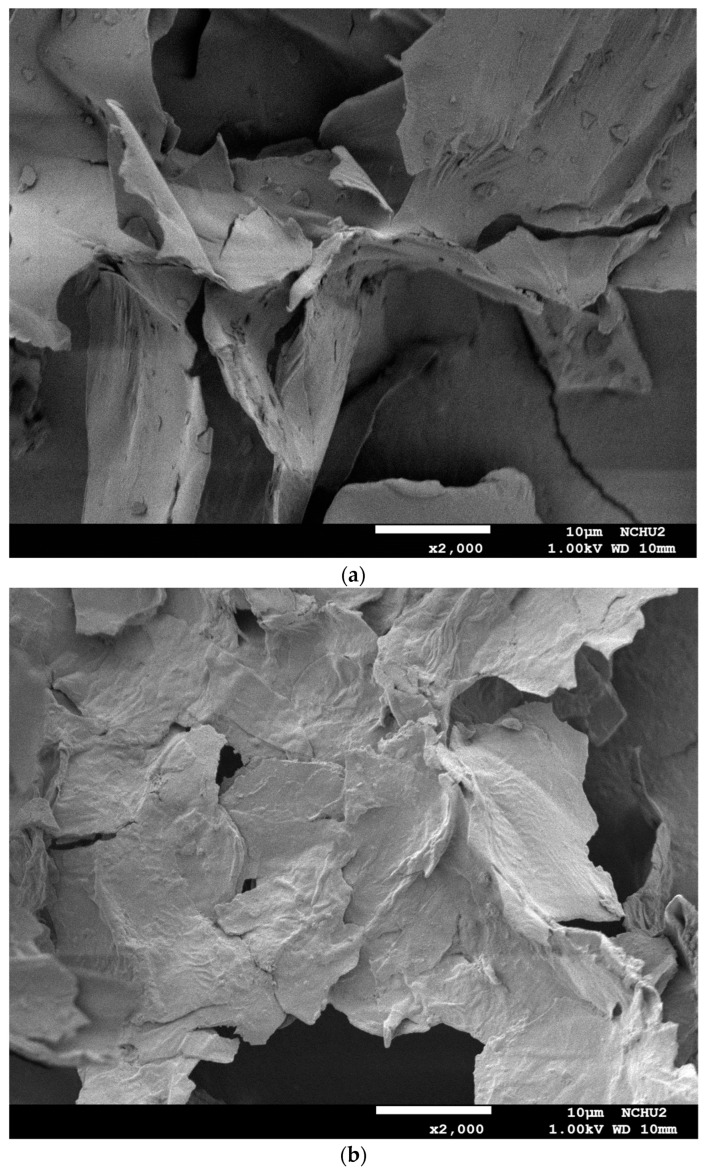

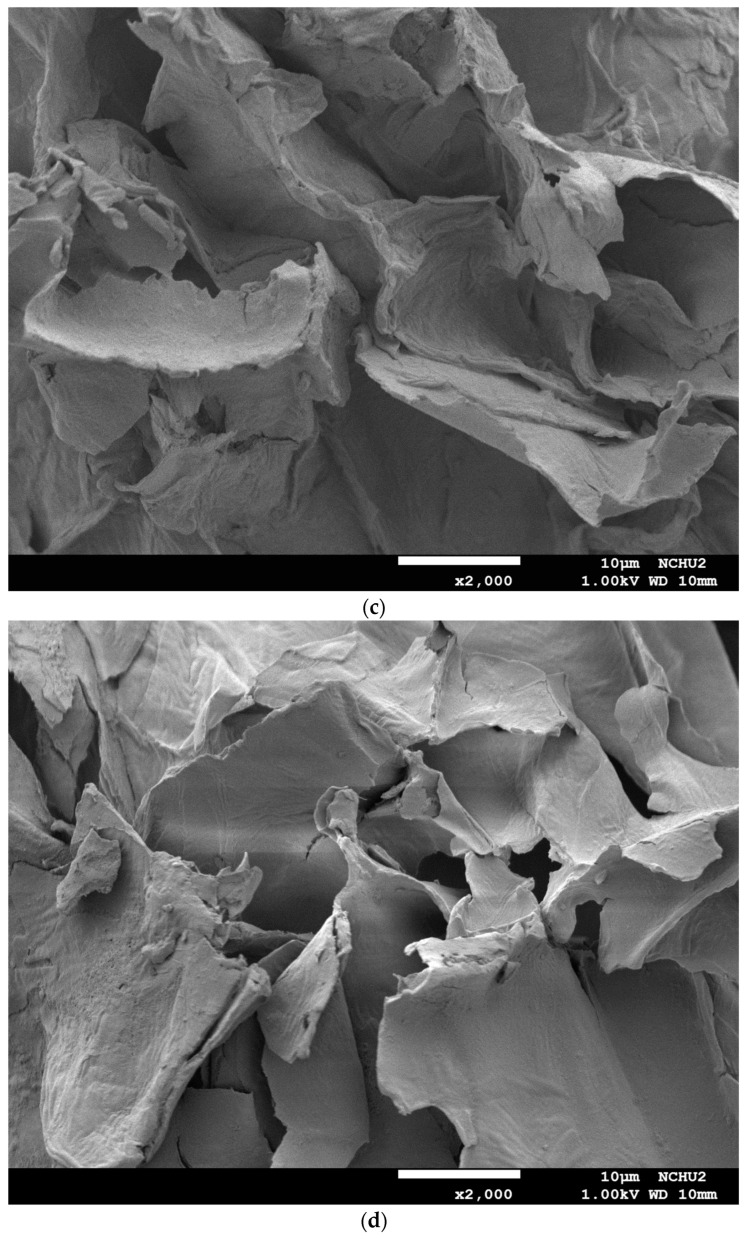
Comparison of FESEM images of dried burdock roots before and after extraction at 50 °C, 1/30 (g/mL-water) of solid-to-liquid ratio, and 10 min of extraction time: (**a**) dried burdock root powder before extraction (×2000); (**b**) extraction residue using 120 rpm of shaking (×2000); (**c**) extraction residue using 40 kHz of ultrasound (×2000); (**d**) extraction residue using 120 kHz of ultrasound (×2000).

The effect of different extraction modes on the microstructures of the burdock roots was verified by observing the surface morphology of the roots before and after extraction using FESEM. Figure 1a is the image of the microstructure of freeze-dried burdock root powder before extraction, presenting with some particulate materials attached to a larger flake structure on the surface. Figure 1b is the image of the extraction residue of burdock roots after 10 min of shaking at 120 rpm. It was found that the particulate materials attached to the surface of extraction residue had disappeared from the shearing effect by shaking, and the flake structure became more flat with some degrees of swelling by the water solvent, so as to facilitate the transport of dissolved compounds. Figure 1c,d show the extraction residue of burdock roots after 10 min of ultrasonic irradiation using 40 and 120 kHz/300 W, respectively. It revealed that after the extraction with ultrasound at 40 or 120 kHz, the flake structure was much more broken with many traces of erosion on the surface, especially having a rougher surface structure when using 40 kHz of ultrasound. This confirmed that the cavitation effect by ultrasound could destroy the original flake structure, increasing the interstices and pores for solvent transport into the internal structure, thus enhancing the extraction efficiency of bioactive compounds.

Table 1 displays the comparison of extraction efficiency of the bioactive compounds, inulin, and antioxidant activity for the extraction modes of 120 rpm of shaking (S-120), 40 kHz/300 W of ultrasound (U-40), and 120 kHz/300 W of ultrasound (U-120) at 50 °C and 1/30 (g-DBR/mL-water) of solid-to-liquid ratio in 10 min of extraction time. As shown in Table 1, the extract yields using water as the solvent were 75.98, 76.04, and 75.81% with S-120, U-40, and U-120, respectively, showing an insignificant difference in extraction yield for the three extraction modes; in addition, the values of TPC in the dried burdock root powder (DBR) with S-120 and U-40 were slightly higher than that with U-120, but with an insignificant difference. Considering that CGA is one of the phenolic compounds, the CGA content in DBR with U-40 was found to be 484.65 μg/g-DBR, which was considerably more than that obtained with U-120 (369.93 μg/g-DBR) and S-120 (176.99 μg/g-DBR). However, the content of cynarin in DBR using U-120 was 376.47 μg/g-DBR, which was higher than that using U-40 (341.54 μg/g-DBR) and S-120 (330.44 μg/g-DBR). The results showed that the ultrasound was able to be used to promote the better extraction of CGA and cynarin than using shaking, and the ultrasonic frequency exhibited a benefit in the selective extraction of CGA and cynarin. Besides, the order of inulin content in DBR was U-120 (42.89% of g-DBR) > U-40 (40.42% of g-DBR) > S-120 (36.49% of g-DBR), but with an insignificant difference. The levels of inulin content in DBR were approximately consistent with the range of values reported in the literature [16,35]. Watanabe et al. (2020) analyzed the amount of inulin/fructan with a K-FRUC Fructan Assay Kit (Megazyme) and found that approximately 50% of the dry weight in *A. lappa* root was inulin/fructan [35]. The variation of inulin content in burdock roots from various regions could be explained by climate conditions, the geographical origin of burdock, harvest time, and the processing conditions [16].

For the antioxidant activity of the extracts obtained in 10 min of extraction, the order of FRAP was U-40 (14.39 mg-FSE/g-DBR) ≥ U-120 (14.00 mg-FSE/g-DBR) > S-120 (12.23 mg-FSE/g-DBR), showing that using U-40 would give the extract with the highest antioxidant activity. It was found that the extraction efficiencies of total phenolic compounds, and the specific bioactive compounds of CGA, cynarin, and inulin from the burdock roots using water as the solvent, varied greatly between using the various ultrasonic frequencies and shaking. Bu and Alheshibri (2021) indicated that the intensity of the energy released during bubble collapse was approximately inversely proportional to the applied ultrasonic frequency, and the bubbles produced at a high frequency were smaller than those produced at a lower frequency [36]. This implied that using 40 kHz of ultrasound to release a higher energy in the medium facilitated the greater solvation of CGA in the solvent from the burdock root tissues than by using 120 kHz of ultrasound, while the solvation of cynarin was promoted by a higher ultrasonic frequency. Furthermore, the low frequency (20–50 kHz) of ultrasound is beneficial for transient cavitation and the high impact results in transient cavitation effects after the bubbles collapse [36]. Nguyen et al. (2017) indicated that the mechanical effect threshold was nearly equal to the cavitation threshold at a frequency less than 98 kHz, and the cavitation threshold was greatly less than mechanical effect threshold at a high frequency [37]. Thus, it was speculated that the variations in TPC, free CGA, and cynarin in the presence of abundant polysaccharide inulin between using 40 and 120 kHz of ultrasound could be attributed to the ultrasonic effect, dominated by the cavitation threshold or mechanical effect threshold.

### 3.2. Effect of Solid-to-Liquid Ratio

Solid-to-liquid ratio is one of the important factors that influence the extraction efficiency, because it affects the yield of the bioactive extracts from the raw materials. Different solid-to-liquid ratios (1/10 to 1/40 g-DBR/mL-water) were assessed for the ultrasound-assisted extraction of bioactive compounds with 40 kHz/300 W of ultrasound at 50 °C in 10 min of extraction time. The results are shown in Table 2. By using water as the solvent, the extract yield, TPC, CGA, cynarin, inulin, and antioxidant activity were all significantly increased when increasing the amount of water for the solid-to-liquid ratio from 1/10 to 1/30. However, the extract yield, TPC, FRAP, inulin, CGA, and cynarin between using 1/30 and 1/40 of solid-to-liquid ratios showed insignificant differences. The higher CGA contents in DBR were 484.65 and 487.33 μg/g-DBR for 1/30 and 1/40 solid-to-liquid ratios, respectively, and the highest cynarin content in DBR was 341.54 μg/g-DBR, obtained by using a 1/30 of solid-to-liquid ratio. Kumar et al. (2021) indicated that the viscosity of the solution at the higher solid-to-liquid ratio (with less solvent) was high enough to increase the difficulty in achieving a cavitation effect, and the lower solid-to-liquid ratio (with more solvent) could increase the contact area between the solvent and the solid material to promote the dissolution of the solute in the solvent, resulting in the ultrasonic intensity acting on the solid material with a more significant effect on fragmentation and erosion [38]. It was speculated that, in the presence of abundant polysaccharide inulin extracted into the aqueous solution, at the higher solid-to-liquid ratio (with less water), the difficulty in achieving the cavitation effect could be increased due to the extraction of inulin, increasing the viscosity of the aqueous solution; when using the lower solid-to-liquid ratio (with more water), the contact area for the dissolution of bioactive compounds from the solid material would be increased, resulting in a more significant cavitation effect of ultrasonic intensity on the solid material. For different solid-to-liquid ratios, the contents of bioactive compounds extracted from burdock roots by using ultrasound would be improved with increasing the amount of water up to a certain point and then slightly changed. Hence, the favorable solid-to-liquid ratio was set at 1/30 (g-DBR/mL-water).

### 3.3. Effect of Temperature

**Figure 2:**
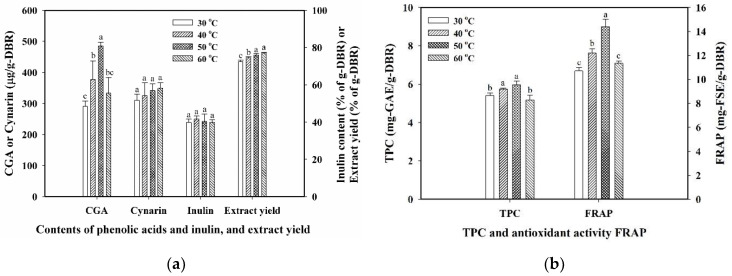
Effect of temperature on (**a**) contents of chlorogenic acid (CGA), cynarin, inulin, and extract yield, and (**b**) total phenolic content (TPC) and ferric-reducing antioxidant power (FRAP). Conditions: 40 kHz/300 W of ultrasound, 10 min of extraction, and 1/30 (g/mL-water) of solid-to-liquid ratio. Data were expressed as mean ± standard deviations from triplicate experiments. Different superscript letters at the same group were significantly different (*p* < 0.05) by the Duncan’s test.

Temperature is the critical factor related to the extraction efficiency of bioactive compounds from plant materials. An increase in temperature can increase the solubility of the solute in the solvent and promote the transport of solute from solid material. It can also reduce the viscosity of the solvent to facilitate the diffusion of the solvent into the internal structure. Various extraction temperatures (30 to 60 °C) were tested for the extraction efficiency of the extract yield, CGA, cynarin, inulin, TPC, and antioxidant activity in the conditions of 40 kHz/300 W of ultrasound, 1/30 (g-DBR/mL-water) of solid-to-liquid ratio, and 10 min. The results are displayed in Figure 2a for CGA, cynarin, inulin, and extract yield, and Figure 2b for TPC and FRAP. Under ultrasound at 40 kHz, the content of free CGA in DBR was significantly increased from 290.75 μg/g-DBR at 30 °C to 484.65 μg/g-DBR at 50 °C, and then decreased to 333.22 μg/g-DBR at 60 °C (Figure 2a). It revealed that the ultrasonic irradiation might induce the degradation, with a greater amount of free CGA bound to the existing abundant inulin, and with more of the free CGA degraded at a higher temperature, due to the local hotspots caused by the cavitation effect or mechanical effect in the liquid medium [38,39]. Moreover, it was also found that the effect of the extraction temperature on the inulin content was insignificant, because of the moderate solubility of inulin in water (≈0.10 at 303 K) [40]. While for cynarin, the content of cynarin in DBR only slightly increased with increasing temperature from 30 to 60 °C with insignificant differences, demonstrating that the severity for the degradation of cynarin at a higher temperature was not so much as that of CGA. In addition, as shown in Figure 2b, the variation of TPC by the increase of temperature also exhibited a similar trend to that of the free CGA content. The antioxidant activity FRAP was significantly increased from 10.70 mg-FSE/g-DBR at 30 °C to 14.39 mg-FSE/g-DBR at 50 °C, then decreased to 11.34 mg-FSE/g-DBR at 60 °C. This trend of increase then decrease in FRAP from 30 to 60 °C corresponded to that of the free CGA content and TPC, showing the contribution of the phenolic compounds to the antioxidant activity.

### 3.4. Effect of Extraction Time

**Figure 3:**
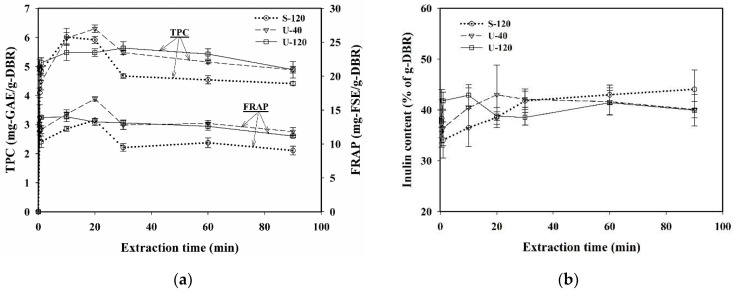
Effect of extraction time on the variations of (**a**) total phenolic content (TPC) and ferric-reducing antioxidant power (FRAP), and (**b**) inulin content. Conditions: 50 °C and 1/30 (g/mL-water) of solid-to-liquid ratio for extraction modes of 120 rpm of shaking (S-120), 40 kHz of ultrasound (U-40), and 120 kHz of ultrasound (U-120). Data were expressed as mean ± standard deviations from triplicate experiments.

The extraction efficiency of polyphenols as well as some specific compounds from plant materials is also affected by the duration of extraction time, due to the interaction between the extracted components and the processing conditions. The comparison of using S-120, U-40, and U-120 for the effect of extraction time on the extraction efficiency of TPC, FRAP, CGA, cynarin and inulin was performed at 50 °C and 1/30 (g-DBR/mL-water) of solid-to-liquid ratio. The results are displayed in Figure 3a for TPC and FRAP, and Figure 3b for the inulin content based on DBR.

As shown in Figure 3a, the amount of TPC in DBR first increased with the increase in extraction time, and then gradually decreased against the extraction time for S-120, U-40, and U-120. The extent of first increasing and then decreasing for the rate of TPC by the increase of extraction time for U-120 was smaller than that for U-40 and S-120. The trends were consistent with the variations of FRAP against the extraction time for the three extraction modes. In addition, when the extraction time was over 30 min, both TPC and FRAP for U-40 were almost equivalent to that for U-120, and were still much greater than that of S-120. The results confirmed the higher extraction efficiency of bioactive compounds by ultrasound than by shaking.

Moreover, for the extraction of inulin using water as the solvent, the effect of time on the extraction of inulin was not much significant. The inulin content was quickly increased to the limiting value for S-120, U-40, and U-120, and the differences between these three extraction modes were obviously insignificant, showing that the extraction of inulin in water was not affected much by using ultrasound or shaking, probably due to inulin being a soluble dietary fiber, having a moderate solubility in water [40].

**Figure 4:**
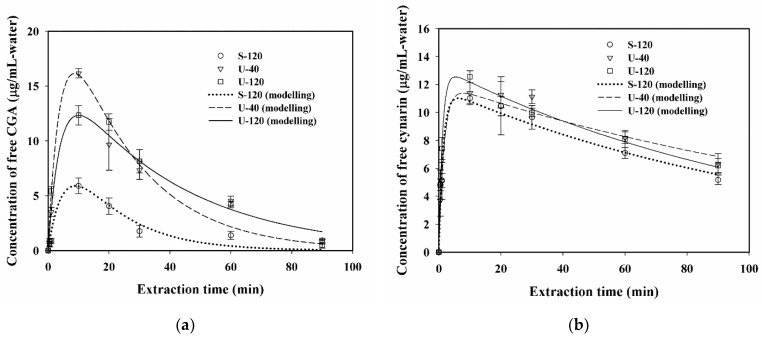
Plots of (**a**) concentration of free chlorogenic acid (CGA) and (**b**) concentration of free cynarin in water versus extraction time, and modelling the correlation of related kinetic parameters. Conditions: 50 °C and 1/30 (g/mL-water) of solid-to-liquid ratio for extraction modes of 120 rpm of shaking (S-120), 40 kHz/300 W of ultrasound (U-40), and 120 kHz/300 W of ultrasound (U-120). Data were expressed as mean ± standard deviations from triplicate experiments. Symbols: experimental data; lines: modelling results.

The plots of the concentration of free CGA and cynarin (μg/mL-water, calculated by multiplying μg/g-DBR with g-DBR/mL-water) in the aqueous solution versus extraction time for S-120, U-40, and U-120 are displayed in Figure 4a,b for free CGA and cynarin, respectively. From the profiles of free CGA and cynarin against extraction time, both the concentrations of free CGA and cynarin first increased at the early time to the maximum value, then decreased at the late time when using S-120, U-40, and U-120, but with different extents of the reducing rate in the period of 20 to 90 min of the extraction. When the duration of extraction was over 20 min, the degradation of free CGA was observed to be more severe than that of cynarin for the three extraction modes. It also revealed that, when extending the extraction time over 20 min, the significant degradation of free CGA in the aqueous solution was suggested to result from the two effects, i.e., a higher severity of thermal degradation and phenolic binding of CGA with the abundant polysaccharide inulin. Such phenomena of thermal degradation and interactions between CGA and polysaccharide inulin were similar to those reported in previous literatures [15,20,41]. For example, Chen et al. (2004) investigated heat treatment on the degradation of CGA in the aqueous solution, and found that the CGA contents remained at about 75 and 67% in the solution when incubated at 98 °C for 20 and 30 min in a water bath, respectively [41], showing that the extent of thermal degradation of CGA increased with increasing time in the aqueous solution. Yi et al. (2022) indicated that the interaction of phenolic binding of CGA on polysaccharides caused significant changes in the aggregation behavior of lotus root polysaccharides [42]. Furthermore, Tomas et al. (2018) investigated the effect of the addition of inulin on the phenolic content of tomato sauces, and found that the addition of inulin to tomato sauce significantly decreased the total phenolic content (57–68%) and total antioxidant capacity (49–61%), probably due to the interaction between inulin and phenolic compounds [43].

Such an interaction can also be related to the soluble or insoluble dietary fiber content. Phan et al. (2015) reported that for different polyphenol classes all bound to cellulose spontaneously and rapidly (occurring within 1 min of contact), the adsorbed amounts increased with increasing the exposure times, which reached up to 60% *w*/*w* of cellulose [44]. In general, the factors that could influence the binding include the polyphenol structure, the number of OH groups, the degree of aggregation of dietary fibers, the size and stability of aggregates, and the conformational organization of polymeric molecules [45]. The interactions between polyphenols and dietary fibers can depend on the hydrophilic or hydrophobic properties of polyphenols and their solubility in water, such as polyphenols with more hydrophilic properties preferring contact with hydrophilic fibers, and these interactions being mainly driven by the hydrogen bonds [45,46]. In this study, CGA, having the relatively hydrophilic quinic acid moiety, was expected to prefer the contact with the soluble dietary fiber inulin during the period of extraction when using water as the solvent.

To further test the stability of free CGA existing in the water extract from burdock root powder, the water extract containing 153.88 ± 5.26 μg/g-BRE (*n* = 3) of CGA was re-dissolved in water in the solid-to-liquid ratio of 1/30 (g-BRE/mL-water) at 50 °C and then sonicated using 40 kHz/300 W of ultrasound for 30 and 60 min. After completing the sonication, the liquid sample was prepared for the measurement of the remaining free CGA using methanol to form the precipitates, which were removed by centrifugation. The results showed that the contents of free CGA remaining in the aqueous solution were less than 10 μg/g-BRE for both 30 and 60 min of sonication. This confirmed that the degradation of free CGA in the aqueous phase was very significant under sonication. Hence, the significant degradation of CGA in the aqueous solution after a certain point of extraction time could be reasonably speculated to result from the thermal degradation and binding with polysaccharide inulin during the extraction using water. Therefore, the choice of a suitable extraction point in time frame was beneficial for the maximum production of free CGA and cynarin in the presence of inulin from burdock roots.

This trend of the CGA and cynarin concentrations increased at an early time and decreased at a late time, which was consistent with that of TPC against extraction time, showing the important contributions of CGA and cynarin to antioxidant activity. Besides, the concentration of free CGA by S-120 was all greatly less than that by U-40 and U-120, as shown in Figure 4a, revealing that the intensity of the shearing effect by shaking at 120 rpm was less than that of cavitation effect and mechanical effect by ultrasound at 40 and 120 kHz.

### 3.5. Kinetic Modeling for Variations of Free CGA and Cynarin

The kinetic model of first-order series reactions was proposed to describe the phenomena of extraction followed by degradation for free CGA and cynarin extracted from burdock roots using water as the solvent. The values of correlated kinetic parameters *k*, *k_d_*, and *C_E_* of CGA and cynarin for S-120, U-40, and U-120 by using Equations (7) and (9) are shown in Table 3. The value of R-square of correlation was above 0.92 for each case, except for the value of 0.8996 for free CGA with S-120. The *k* value for free CGA with S-120 (0.1876 min^−1^) was much less than that U-40 (0.2439 min^−1^) and U-120 (0.2540 min^−1^), indicating that the cavitation effect by ultrasound was more intensified than the shearing effect by shaking for the extraction of CGA. In addition, using U-40 would give the highest *C_E_* (23.409 µg/mL-water) for the extraction, due to the higher intensity of U-40. For cynarin, the order of *k* value was U-120 (0.8689 min^−1^) > S-120 (0.7248 min^−1^) > U-40 (0.5924 min^−1^), but the use of U-40 gave the smallest *k_d_* value (0.006329 min^−1^), revealing that a lower extraction rate coefficient for cynarin could be accompanied with a lower rate of degradation with U-40. However, the degree of degradation for cynarin was obviously smaller than that for CGA, implying that the ultrasonic frequency and processing time might be applied to selectively extract CGA and cynarin from the burdock roots.

## 4. Conclusions

In this study, the ultrasound-assisted extraction was applied in the extraction of polyphenols and polysaccharides from burdock roots of the Yanagawarisou variety using water as a green solvent. The effect of ultrasound with various frequencies on the extraction efficiency was analyzed for TPC, CGA, cynarin, inulin, and the antioxidant activity of the extract, and shaking extraction was also performed for comparison. The solid-to-liquid-ratio, temperature, and extraction time presented as the important factors for the ultrasound-assisted extraction with 40 kHz/300 W of ultrasound. The ultrasound-assisted extraction with frequencies of 40 and 120 kHz showed a distinct selectivity for the extraction of CGA and cynarin in the presence of polysaccharide inulin, and gave higher extraction efficiencies than the shaking extraction. The profiles of increase and then decrease in free CGA and cynarin concentrations against time suggested the possibility of their degradation and interaction with the abundant inulin in the aqueous solution. Besides, the test for the stability of free CGA existing in the water extract from burdock roots confirmed the significant degradation of free CGA in the aqueous solution after a certain duration of sonication. The kinetic model, considering extraction followed by degradation, was proposed to describe the variations of free CGA and cynarin in the aqueous solution, and the kinetic parameters were successfully correlated. This study provides an effective method to extract CGA, cynarin, and inulin from burdock roots, and the choice of a suitable extraction point in the time frame was beneficial for the maximum extraction of CGA and cynarin from burdock roots. Furthermore, an extract containing antioxidants, from burdock roots, can be served as a nutritional supplement in food application.

## Figures and Tables

**Table 1:** Comparison of total phenolic content (TPC), ferric-reducing antioxidant power (FRAP), inulin, chlorogenic acid (CGA), and cynarin in the dried burdock root powder (DBR) between using ultrasound at 40 kHz/300 W (U-40), ultrasound at 120 kHz/300 W (U-120), and shaking at 120 rpm (S-120).

Items *	Extraction Mode		
	S-120	U-40	U-120
Extract yield (% of g-DBR)	75.98 ± 0.63 ^a^	76.04 ± 0.64 ^a^	75.81 ± 1.12 ^a^
TPC (mg-GAE/g-DBR)	6.01 ± 0.29 ^a^	5.97 ± 0.20 ^a^	5.48 ± 0.29 ^a^
FRAP (mg-FSE/g-DBR)	12.23 ± 0.36 ^b^	14.39 ± 0.64 ^a^	14.00 ± 0.70 ^a^
Inulin (% of g-DBR)	36.49 ± 3.74 ^a^	40.42 ± 3.94 ^a^	42.89 ± 2.09 ^a^
CGA (μg/g-DBR)	176.99 ± 21.51 ^c^	484.65 ± 12.79 ^a^	369.93 ± 26.49 ^b^
Cynarin (μg/g-DBR)	330.44 ± 14.03 ^b^	341.54 ± 21.72 ^b^	376.47 ± 12.94 ^a^

* Conditions: 1/30 (g/mL-water) of solid-to-liquid ratio, 10 min of extraction time, 50 °C. Data were expressed as mean ± standard deviations from triplicate experiments. Different superscript letters at the same row were significantly different (*p* < 0.05) by the Duncan’s test.

**Table 2:** Effect of solid-to-liquid ratio on extract yield, total phenolic content (TPC), ferric-reducing antioxidant power (FRAP), inulin, chlorogenic acid (CGA), and cynarin in dried burdock root powder (DBR).

Items *	Solid-to-Liquid Ratio (g-DBR/mL-Water)			
	1/10	1/20	1/30	1/40
Extract yield (% of g-DBR)	60.20 ± 2.05 ^c^	70.42 ± 0.47 ^b^	76.04 ± 0.64 ^a^	77.37 ± 2.23 ^a^
TPC (mg-GAE/g-DBR)	3.77 ± 0.17 ^c^	5.09 ± 0.08 ^b^	5.97 ± 0.20 ^a^	6.04 ± 0.15 ^a^
FRAP (mg-FSE/g-DBR)	7.50 ± 0.85 ^c^	9.69 ± 0.69 ^b^	14.39 ± 0.64 ^a^	13.37 ± 0.08 ^a^
Inulin (% of g-DBR)	25.63 ± 4.90 ^b^	28.18 ± 1.96 ^b^	40.42 ± 3.94 ^a^	37.20 ± 4.12 ^a^
CGA (μg/g-DBR)	330.45 ± 29.84 ^c^	393.28 ± 25.43 ^b^	484.65 ± 12.79 ^a^	487.33 ± 12.49 ^a^
Cynarin (μg/g-DBR)	213.03 ± 20.41 ^c^	288.12 ± 18.06 ^b^	341.54 ± 21.72 ^a^	329.48 ± 24.01 ^a^

* Conditions: 40 kHz/300 W of ultrasound, 50 °C, 10 min of extraction time. Data were expressed as mean ± standard deviations from triplicate experiments. Different superscript letters at the same row were significantly different (*p* < 0.05) by the Duncan’s test.

**Table 3:** Correlated values of *k*, *k_d_*, and *C_E_* using the kinetic model of extraction followed by degradation for the extraction of chlorogenic acid (CGA) and cynarin from dried burdock root powder.

Extraction Mode *	Bioactive Compound	*k* (min^−1^)	*k_d_* (min^−1^)	*C_E_* (µg/mL-Water)	R-Square
S-120	CGA	0.1876	0.05982	10.073	0.8996
U-40	CGA	0.2439	0.04289	23.409	0.9220
U-120	CGA	0.2540	0.02597	15.988	0.9502
S-120	Cynarin	0.7248	0.008315	11.601	0.9669
U-40	Cynarin	0.5924	0.006329	11.957	0.9804
U-120	Cynarin	0.8689	0.008682	13.146	0.9925

* Conditions: 1/30 (g/mL-water) of solid-to-liquid ratio, 50 °C. S-120: 120 rpm of shaking; U-40: 40 kHz/300 W of ultrasound; U-120: 120 kHz/300 W of ultrasound.

## Data Availability

Data are contained within the article.
